# Emerging role of deubiquitination modifications of programmed death-ligand 1 in cancer immunotherapy

**DOI:** 10.3389/fimmu.2023.1228200

**Published:** 2023-06-21

**Authors:** Yao Jiang, Kai Hong, Yingchao Zhao, Kai Xu

**Affiliations:** ^1^Cancer Center, Union Hospital, Tongji Medical College, Huazhong University of Science and Technology, Wuhan, China; ^2^Institute of Radiation Oncology, Union Hospital, Tongji Medical College, Huazhong University of Science and Technology, Wuhan, China; ^3^Department of Medical Ultrasound, Tongji Hospital, Tongji Medical College, Huazhong University of Science and Technology, Wuhan, China; ^4^Department of Otolaryngology-Head and Neck Surgery, Tongji Hospital, Tongji Medical College, Huazhong University of Science and Technology, Wuhan, China

**Keywords:** deubiquitinating enzymes, deubiquitination, cancer immunotherapy, post-translational modification, programmed death-ligand-1 (PD-L1)

## Abstract

Immune evasion is essential for carcinogenesis and cancer progression. Programmed death-ligand 1 (PD-L1), a critical immune checkpoint molecule, interacts with programmed death receptor-1 (PD-1) on immune cells to suppress anti-tumor immune responses. In the past decade, antibodies targeting PD-1/PD-L1 have tremendously altered cancer treatment paradigms. Post-translational modifications have been reported as key regulators of PD-L1 expression. Among these modifications, ubiquitination and deubiquitination are reversible processes that dynamically control protein degradation and stabilization. Deubiquitinating enzymes (DUBs) are responsible for deubiquitination and have emerged as crucial players in tumor growth, progression, and immune evasion. Recently, studies have highlighted the participation of DUBs in deubiquitinating PD-L1 and modulating its expression. Here, we review the recent developments in deubiquitination modifications of PD-L1 and focus on the underlying mechanisms and effects on anti-tumor immunity.

## 1 Introduction

Immune evasion is essential for carcinogenesis and cancer progression. Cancer cells have developed multiple mechanisms to evade immune surveillance, including reducing immunogenicity, limiting antigen recognition, inducing T cell exhaustion, and expressing inhibitory immune checkpoint proteins (1). Among these checkpoint molecules, programmed death-ligand 1 (PD-L1) is one of the most critical players. PD-L1 interacts with programmed death receptor-1 (PD-1) on immune cells, including T cells, dendritic cells, macrophages, and natural killer (NK) cells to restrain anti-tumor immunity (2). Elevated PD-L1 expression has been observed in multiple cancers including cervical cancer, non-small cell lung cancer (NSCLC), and hepatocellular cancer (3). Currently, PD-1/PD-L1-targeting treatments have significantly affected cancer treatment approaches, and PD-L1 expression has emerged as an indicator for the selection of patients who are more likely to benefit from PD-1/PD-L1 inhibitors (4, 5). Therefore, exploring PD-L1 regulatory mechanisms is of great importance.

The expression of PD-L1 is modulated at various levels, including epigenetic, transcriptional, post-transcriptional, and post-translational mechanisms (3, 6–8). Deubiquitination and ubiquitination are among the most important post-translational modifications, which dynamically control protein degradation and stability, thereby influencing cellular processes. Deubiquitination, mediated by deubiquitinating enzymes (DUBs), involves the covalent cleavage of conjugated monoubiquitin or polyubiquitin chains from various substrates (9, 10). To date, approximately 100 DUBs have been discovered. Based on their structural homology, DUBs can be classified into seven categories as follows: ubiquitin-specific proteases (USPs), otubain proteases (OTUs), and JAB1/MPN/Mov34 metalloenzymes (JAMMs), ubiquitin C-terminal hydrolases, Machado-Joseph disease proteases, MIU-containing novel DUB family proteases, and Zn-finger and UFSP domain proteins. With the exception of JAMMs, all of these DUBs belong to the cysteine protease family.

**Figure 1:**
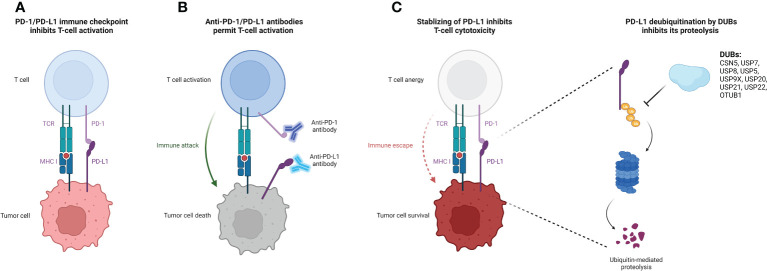
Deubiquitination of PD-L1 protein by DUBs causes increased PD-L1 stability and suppressed T-cell cytotoxicity. **(A)** Binding of PD-L1 on the tumor cell surface to their receptor PD-1 on the T cell surface releases the immune suppression signal, thereby inhibiting T cell activation and cytotoxicity. **(B)** The administration of specific antibodies to PD-L1/PD-1 reverses the T cell activation suppression signal, facilitating for the immune attack form of cytotoxic T cells to target tumor cells. **(C)** DUBs of PD-L1 stabilize PD-L1 and protect it from ubiquitin-mediated proteolysis, promoting tumor cell immune escape from T cell attack. The figure was created with Biorender.com. PD-L1, programmed death-ligand-1; DUB, deubiquitinating enzyme; MHC-I, major histocompatibility complex class I; PD-1, programmed death receptor-1; TCR, T cell receptor.

DUBs have been extensively studied in a variety of cellular activities including cell proliferation, apoptosis, cell cycle control, and adaptive immune response (11). Several DUBs have been demonstrated to deubiquitinate PD-L1 and regulate its expression in cancer (7) (**Figure 1**). Here, we provide a comprehensive review of the recent advancements in deubiquitination modifications of PD-L1, focusing on their impact and the underlying mechanisms related to anti-tumor immunity (**Table 1**).

**Table 1:** Major deubiquitinating enzymes (DUBs) of PD-L1 and their biological effects in cancer.

DUBs	Categories	Mechanism	Types of cancer	Related molecules	Effects	References
CSN5	JAMM	Removes K48-linked ubiquitin on PD-L1	Breast cancer	NF-κB p65, GATA-AS1	Inhibits anti-tumor function of T cells	(12, 13)
			Hepatocellular cancer	GOLM1	Inhibits CD8^+^ T cell cytotoxicity	(14)
			Pancreatic cancer	PDIA6	Inhibits NK cell function	(15)
			Colorectal cancer	CCL5, NF-κB p65	Inhibits CD8^+^ T cell cytotoxicity	(16)
			NSCLC	BBR	Inhibits intratumor T cell infiltration	17)
USP7	USP	Stabilizes PD-L1 through deubiquitination	Glioma	N/A	Inhibits CD8^+^ T cell cytotoxicity	(18)
			Gastric cancer	N/A	Inhibits T cell mediated cytotoxicity and tumor cell proliferation	(19)
USP8	USP	Removes K63-linked ubiquitin on PD-L1	Multiple cancer types	TRAF6, NF-κB	Inhibits MHC-I-dependent antigen presentation	(20)
		Stabilizes PD-L1 through deubiquitination	Pancreatic cancer	N/A	Inhibits CD8^+^ T cell cytotoxicity	(21)
			NSCLC	LncRNA SNHG12, HuR	Inhibits CD8^+^ T cell cytotoxicity	(22)
USP5	USP	Stabilizes PD-L1 through deubiquitination	NSCLC	N/A	Inhibits CD8^+^ T cell cytotoxicity	(23)
USP9X	USP	Stabilizes PD-L1 through deubiquitination	Oral cancer	N/A	Inhibits T cell cytotoxicity	(24)
USP20	USP	Stabilizes PD-L1 through deubiquitination	Breast cancer	TINCR	N/A	(25)
USP21	USP	Removes K48-linked ubiquitin on PD-L1	Lung cancer	N/A	N/A	(26)
			Colorectal cancer	STAT3, Foxp3	Promotes Treg cell function	(27)
USP22	USP	Stabilizes PD-L1 through deubiquitination	NSCLC	CSN5	Inhibits T cell cytotoxicity	(28)
			Liver cancer	N/A	Inhibits intratumor T cell infiltration	(29)
OTUB1	OTU	Removes K48-linked ubiquitin on PD-L1	Breast cancer	N/A	Inhibits T cell cytotoxicity	(30)
			NSCLC	PKP3	Inhibits CD8^+^ T cell infiltration	(31)

PD-L1, programmed death-ligand-1; DUBs, deubiquitinating enzymes; USP, ubiquitin-specific proteases; OTU, otubain proteases; JAMM, JAB1/MPN/Mov34 metalloenzymes; NF-κB, nuclear factor κB; GATA-AS1, GATA binding protein 3 antisense RNA 1; CSN5, The constitutive photomorphogenesis 9 (COP9) signalosome 5; GOLM1, Golgi membrane protein 1; PDIA6, protein disulfide isomerase family A member 6; CCL5, C-C motif chemokine ligand 5; NSCLC, non-small cell lung cancer; BBR, berberine; N/A, not applicable; TRAF6, TNF receptor associated factor 6; LncRNA SNHG12, lncRNA small nucleolar RNA host gene 12; HuR, human antigen R; TINCR, tissue differentiation inducing non-protein coding RNA; STAT3, signal transducer and activator of transcription 3; Foxp3, forkhead box P3; PKP3, plakophilin 3; MHC-I, major histocompatibility complex class I; NK, natural killer.

## 2 Emerging progress on the regulation of PD-L1 by DUBs in cancer

### 2.1 CSN5

The constitutive photomorphogenesis 9 signalosome 5 (CSN5) contains a conserved JAMM motif, which belongs to the JAMM subfamily (32). On one hand, CSN5 could directly interact with a variety of molecules, including c-Jun and p53, thereby influencing tumor proliferation (12). On the other hand, as a deubiquitinase, CSN5 deubiquitinates PD-L1, promoting tumor progression and immune escape (12). One study demonstrated that the activation of nuclear factor κB (NF-κB), further transactivates CSN5, leading to PD-L1 deubiquitination and stabilization (12). Moreover, PD-L1 and CSN5 expression levels are positively correlated in breast cancer (12). The application of a CSN5 inhibitor, curcumin, results in PD-L1 destabilization, increases the cytotoxic activity of T cells, and synergizes with anti-cytotoxic T-lymphocyte associated protein 4 antibodies (12).

Multiple mechanisms regulate CSN5-mediated PD-L1 deubiquitination. Golgi membrane protein 1 upregulates the expression of PD-L1 in hepatocellular cancer cells through the CSN5-mediated deubiquitination of PD-L1, leading to the suppression of CD8^+^ T cells (14). Ma et al. reported that protein disulfide isomerase family A member 6 interacts with CSN5 and promotes the deubiquitination of PD-L1 in pancreatic cancer cells (15). In colorectal cancer, macrophages-derived C-C motif chemokine ligand 5 (CCL5) promotes the activation of NF-κB p65 activation, which binds to the *CSN5* promoter, increases CSN5 expression, and upregulates PD-L1 protein level (16). In triple negative breast cancer, long non-coding RNA GATA binding protein 3 antisense RNA 1 stabilized PD-L1 *via* the miR-676-3p/CSN5 axis (13). Interestingly, berberine, an established anti-inflammatory drug, interacts with CSN5 and inhibits CSN5/PD-L1 interaction, resulting in PD-L1 ubiquitination (17).

### 2.2 USP7

USP7 is a deubiquitinase that contains a USP domain (33), and is aberrantly expressed in several human cancers. USP7 mediates cell cycle control, tumor growth, chemoresistance, and tumor immunity by regulating multiple cellular signaling pathways, including the p53 and Wnt pathways (33, 34).

Regulatory T cells (Tregs) suppress the activity of effector T cells and promote immune escape (35). Moreover, Tip60, a histone acetyltransferase, promotes the acetylation and dimerization of the key transcription factor, forkhead box P3 (Foxp3), and regulates the activity of Tregs (35). A previous study discovered that USP7 directly deubiquitinates Foxp3 and stabilizes it. Further, USP7 depletion disrupts the immunosuppressive functions of Tregs *in vivo* (36). Another investigation revealed that USP7 is a deubiquitinase of both Tip60 and Foxp3, which enhances Tregs functions by increasing protein abundance (37).

Emerging studies have demonstrated that USP7 expression correlates with PD-L1 levels in cancer (18, 38). One recent investigation observed the overexpression of USP7 and PD-L1 proteins in glioma (18). USP7 mediates the deubiquitination of PD-L1, leading to increased PD-L1 expression. Abrogated USP7 expression promotes CD8^+^ T cell proliferation, elevates tumor necrosis factor (TNF) alpha and interferon gamma (IFN-γ) levels, and inhibits glioma cell immune evasion, which can be reversed by PD-L1 overexpression (18). Similarly, USP7 expression is upregulated and positively associated with PD-L1 in gastric cancer. Silencing USP7 decreases PD-L1 expression on cell surfaces, and augments the T cell-mediated killing of cancer cells (19). However, the regulatory relationship between USP7 and PD-L1 appears to be context-dependent. A negative association between the USP7 and PD-L1 expression in lung adenocarcinoma was revealed using The Cancer Genome Atlas data (38). In addition, the targeted inhibition of USP7 significantly increases PD-L1 protein levels in both cancer cells and the tumor microenvironment. Furthermore, abrogated USP7 expression inhibits the M2 macrophages transformation and their function, and promotes IFN-γ^+^CD8^+^ T cells infiltration, augmenting anti-tumor immunity. Additionally, a USP7 inhibitor, P5091, has shown a synergistic anti-tumor effect with a PD-1 inhibitor *in vivo* (38, 39).

### 2.3 USP8

Increasing evidence suggests that USP8 expression is upregulated, which stabilizes multiple oncogenes, in various cancers (22, 40). Additionally, USP8 is involved in T cell development and homeostasis. It is also essential for thymocyte maturation, proliferation, and the suppressive function of Treg cells on γδ T cells. Mechanistically, USP8 interacts with Gads and 14-3-3β, forming a complex with the T cell receptor (TCR)−CD28 cluster upon stimulation. Subsequently, USP8 is degraded *via* a caspase-dependent pathway, leading to the downregulation of interleukin-7 receptor subunit alpha (IL-7Rα) levels through the Forkhead box protein O1−IL-7Rα axis (41). Another study demonstrated that USP8 deubiquitinates and increases the expression of the type II transforming growth factor-β receptor (TβRII) in tumor-derived extracellular vesicles (TEVs). The inhibition of USP8 reduces the abundance of TβRII^+^ circulating TEVs and prevents CD8+ T cell exhaustion (42).

In a screening study performed by Xiong et al., it was revealed that DUBs-IN-2, a USP8 inhibitor, significantly increases PD-L1 protein levels in multiple cancer cell lines (20). Furthermore, a negative association between USP8 and PD-L1 was confirmed in lung squamous cancer tissues. Mechanistically, USP8 specifically removes K63-linked ubiquitination, but promotes the K48-linked ubiquitination of PD-L1, which finally promotes PD-L1 degradation. Furthermore, by deubiquitinating the K63-linked modification of TNF receptor associated factor 6 (TRAF6), USP8 up-regulates the expression of most genes in the major histocompatibility complex class I pathways, which limits the NF-κB signaling pathway and inhibits the immune response and antigen presentation. A USP8 inhibitor synergizes with anti-PD-1/PD-L1 treatments, dramatically inhibits tumor growth, and improves survival rates in mouse colon cancer models (20). Conversely, a recent study showed that the expression levels of USP8 and PD-L1 are positively correlated in pancreatic cancer. USP8 deficiency decreases PD-L1 protein abundance by promoting PD-L1 ubiquitination-mediated degradation. Moreover, a combined strategy comprising a USP8 inhibitor and PD-L1 inhibitor decreases tumor growth and enhances CD8^+^ T cell mediated killing of cancer cells (21).

### 2.4 USP5

USP5 belongs to the USP subfamily and can specifically recognize unconjugated polyubiquitin and cleave ubiquitin linkages. USP5 participates in multiple cellular procedures, including inflammatory responses (43, 44). The NLR family pyrin domain-containing 3 (NLRP3) inflammasome is critical for defense against microbial pathogens, and its dysregulation is implicated in various inflammatory diseases. Notably, USP5 is involved in regulating NLRP3 inflammasome activity, unrelated to its DUBs function. Mechanistically, USP5 acts as a pivotal scaffold protein recruiting a specific E3 ligase to NLRP3, promoting its ubiquitination and autophagic degradation. In addition, in alum-induced peritonitis mouse models, the overexpression of USP5 reduces interleukin 1 beta (IL-β) levels and polymorphonuclear infiltration (45). Furthermore, recent studies have demonstrated that USP5 directly deubiquitinates and stabilizes PD-L1. In NSCLC tissues, elevated USP5 expression correlates with PD-L1 expression, indicating of unfavorable clinical outcomes. Moreover, the inhibition of USP5 suppresses tumor growth *in vivo* by downregulating PD-L1 expression (23).

### 2.5 USP9X

USP 9, X-linked (USP9X) is a positive regulator of the TCR signaling pathway. Silencing of USP9X *in vivo* suppresses T-cell growth, cytokine production, and the differentiation of T helper (Th) cells, without affecting T-cell survival and the development of specific T-cell populations in the thymus. Moreover, *USP9X* knockdown in human and mouse T-cell lines attenuates the TCR signaling-mediated activation of NF-κB through the deubiquitination of Bcl10 (46). *USP9X* knockout also results in a proliferation defect in both CD4^+^and CD8^+^ T cells, impairs the development of T cells in the thymus, and downregulates proximal TCR signaling. *In vivo* studies demonstrated that the T cell-specific knockout of *Usp9x* elevates PD-1-expressing T cell populations, leading to the incidence of specific autoimmune disease (47). In B lymphocytes, USP9X is necessary for the kinase activity of protein kinase C beta after B cell antigen receptor-dependent activation (48). In a model of sepsis with liver injury, USP9X promotes CD8^+^ T cells dysfunction in the liver through the inhibition of autophagy, which can be reversed by the conditional depletion of mechanistic target of rapamycin (49). Moreover, USP9X directly binds PD-L1, and USP9X reduces PD-L1 ubiquitination and increases its protein abundance. Additionally, a positive association was found between USP9X and PD-L1 expression in oral cancer (24).

### 2.6 USP20

USP20 has been linked to antiviral response, metabolic disease, neuroinflammation, and tumor progression (50–52). Tax is a viral oncoprotein which persistently activates NF-κB signaling and causes adult T cell leukemia. Through deubiquitination of TRAF6 and Tax, USP20 suppresses activation of NF-κB signaling and inhibits proliferation of leukemia cells (53).

A recent study demonstrated that USP20 interacts with PD-L1 and deubiquitinates it, which can be regulated by a long non-coding RNA, tissue differentiation inducing non-protein coding RNA (TINCR). Mechanistically, LncRNA TINCR acts as a competing endogenous RNA, which promotes stability of USP20 mRNA and upregulates the expression of PD-L1 in breast cancer (25).

### 2.7 USP21

Numerous studies have implicated USP21 in regulating cancer cell stemness, tumor growth, and metastasis (26, 54). In regard to immune regulation, Yang et al. have demonstrated a direct interaction between USP21 and PD-L1, whereby USP21 removes polyubiquitin chains from PD-L1, leading to its stabilization. Notably, the expression of USP21 is upregulated in lung cancer tissues, showing a positive association with PD-L1 protein levels (26). Additionally, USP21 has a role in regulating Treg cell functions (27). Li et al. demonstrated that USP21 suppresses the transformation of Th1-like Treg cells by deubiquitinating and stabilizing Foxp3. Mouse models of *Usp21* depletion in Tregs exhibit spontaneous T cell activation and the expanded transformation of Tregs toward the Th1-like phenotype (55). Furthermore, emerging evidence suggests that USP21 suppresses antiviral responses in various immune cell types, including mouse embryonic fibroblasts and bone marrow-derived dendritic cells. This is achieved through the binding and deubiquitination of retinoic acid-inducible gene-I, which restricts type-I interferon production and antiviral immune defense (56). Notably, *Usp21*-knockout mice display enhanced resistance to vesicular stomatitis virus infection with increased production of interferons (56).

### 2.8 USP22

USP22, of which expression levels are elevated in various cancer types, is correlated with disease progression and an unfavorable prognosis (57, 58). This DUB plays a critical role in regulating PD-L1 stability. On one hand, USP22 directly deubiquitinates PD-L1. On the other hand, USP22 interacts with CSN5 and deubiquitinates it, thereby facilitating the interaction between PD-L1 and CSN5 (28). Moreover, USP22 and PD-L1 protein levels are positively correlated in NSCLC samples. The inhibition of USP22 enhances the cytotoxicity of T cells and reduces tumor growth (28). Another study revealed that USP22 interacts with the C terminus of PD-L1 and deubiquitinates it. In mouse models of hepatocellular carcinoma, knockout of *Usp22* increases the infiltration of tumor-infiltrating lymphocytes, augments anti-tumor immunity, and synergizes with anti-PD-L1 treatments and chemotherapy (29). Furthermore, USP22 plays a part in regulating the tumor microenvironment. The knockout of *USP22* in pancreatic ductal adenocarcinoma cells results in reduced myeloid cells infiltration and increased tumor infiltration of NK cells and T cells, leading to a synergistic response with combined immunotherapy (59). USP22 is also involved in regulating invariant NK T (iNKT) cells. USP22 suppression inhibits the development of iNKT cells, and attenuates iNKT1 and iNKT17 cell differentiation, while favoring iNKT2 polarization (60).

### 2.9 OTUB1

OTUB1, a member of the OTU superfamily, exhibits a preference for deubiquitinating K-48 and K-63 ubiquitin chains. Its involvement in cancer development and progression has also been observed (61). Extensive research has highlighted the role of OTUB1 in modulating immune cell responses. Depletion of *OTUB1* activates NK cells and CD8^+^ T cells, leading to increased tumor infiltration of NK cells, DCs and T cells. Additionally, *OTUB1* depletion enhances the cytokine production and the proliferation of CD4^+^ T cells (62, 63).

Moreover, OTUB1 has been reported to specifically interact with of PD-L1, wherein it removes the K48-linked ubiquitin chain from PD-L1 and stabilizes it. Functionally, *OTUB1* depletion decreases PD-L1 expression, and increases the cytotoxicity of human peripheral blood mononuclear cells against tumor cells. The expression of OTUB1 is positively correlated with PD-L1 expression in breast cancer samples. Furthermore, OTUB1 depletion increases CD8^+^ T cell infiltration, elevates serum IFN-γ, and augments anti-tumor immune responses in mouse models (30). Liu et al. reported that circIGF2BP3 acts competitively to upregulate plakophilin 3 (PKP3) expression, which further stabilizes OTUB1 mRNA. CircIGF2BP3/PKP3 suppression synergized with anti-PD-1 treatment in mouse models of lung cancer (31).

## 3 Discussion

Over the past few decades, the application of anti-PD-1/PD-L1 treatments has significantly improved the clinical prognoses of patients with cancer. Nevertheless, the clinical response to single-agent anti-PD-1/PD-L1 antibody therapy is limited to only a subset of patients (64, 65). Combinatorial treatments comprising anti-PD-1/PD-L1 antibodies with antiangiogenic drugs, chemotherapy, and targeted therapy have resulted in more promising clinical outcomes (66). As described above, several DUBs are participated in deubiquitination modifications of PD-L1, and regulated its expression. Thus, developing small-molecule inhibitors targeting these DUBs and the combination therapy represent an attractive therapeutic strategy.

However, despite the importance of these reported DUBs, there are still questions remain to be elucidated. One such question is determining which specific DUB plays the predominant role in regulating PD-L1 expression within a particular type of cancer. Second, although some studies have demonstrated synergistic efficacy of DUBs inhibitors with anti-PD-1/PD-L1 treatments, further exploration through clinical trials is needed to validate these findings and assess their potential for clinical application. In recent years, multiple selective inhibitors of DUBs, including inhibitors of USP7, USP8, and USP9X, have been developed (11). Accordingly, further investigation of these inhibitors in clinical trials is required.

## Author contributions

All authors contributed to the article and approved the submitted version.
